# Supplementary figures and images for: Nascent evolution of recombination rate differences as a consequence of chromosomal rearrangements
Source: PLoS Genet. 2023 Aug 7;19(8):e1010717. doi: 10.1371/journal.pgen.1010717 (PMC10434929; doi:10.1371/journal.pgen.1010717)

Marey map (Catalan)

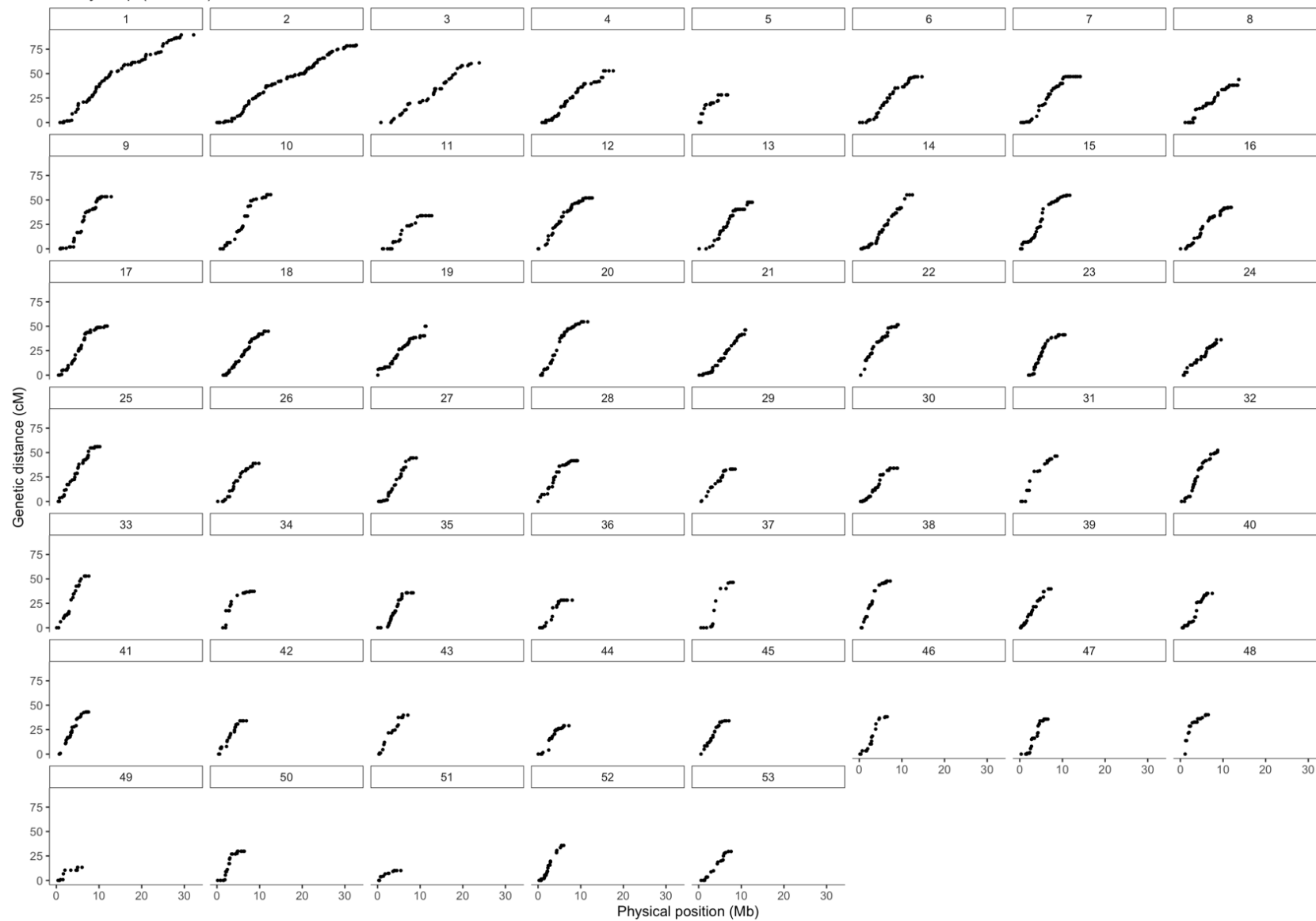

Marey map (Swedish)

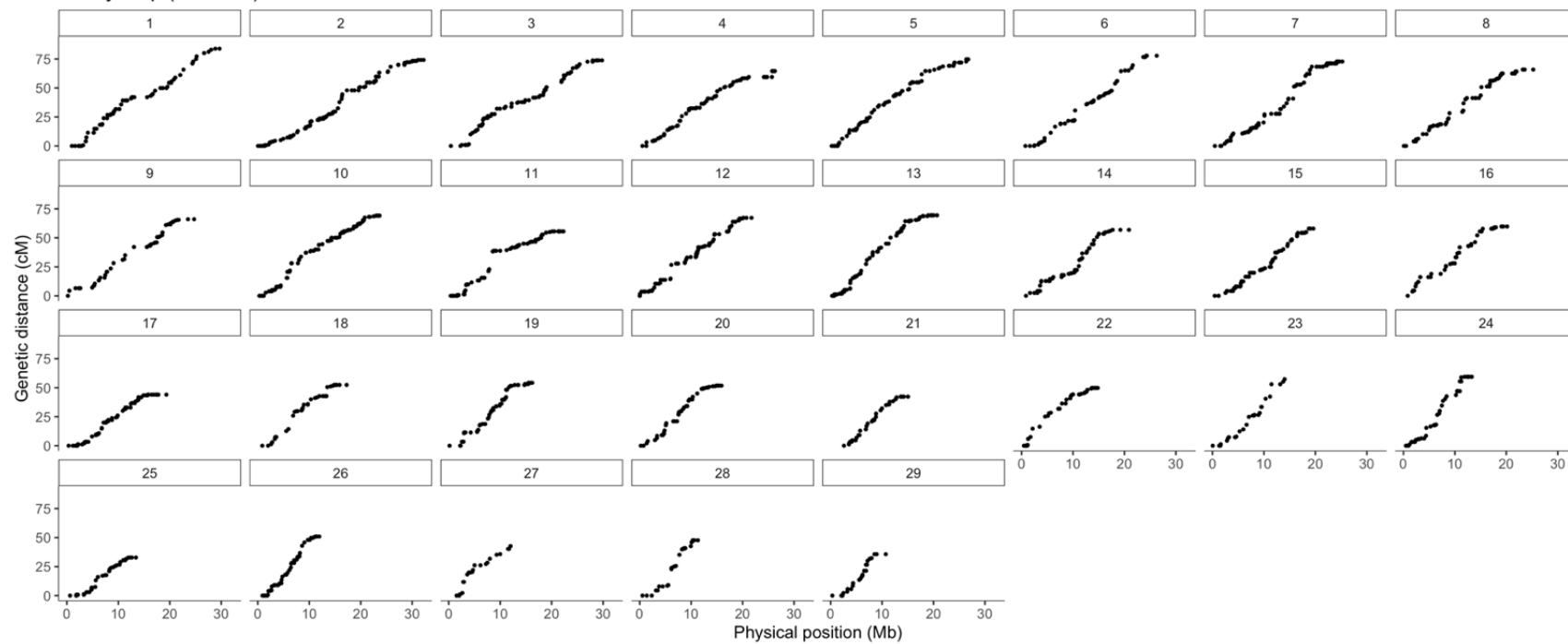

Supplement: S1 Fig — Marey maps for the Catalan (n = 53, top) and the Swedish (n = 29, bottom) population of L. sinapis. Genetic distances are given on the y-axes (cM) and physical positions along each respective chromosome on the x-axes (Mb). (PDF) [file pgen.1010717.s001.pdf]

Recombination rate (Catalan)

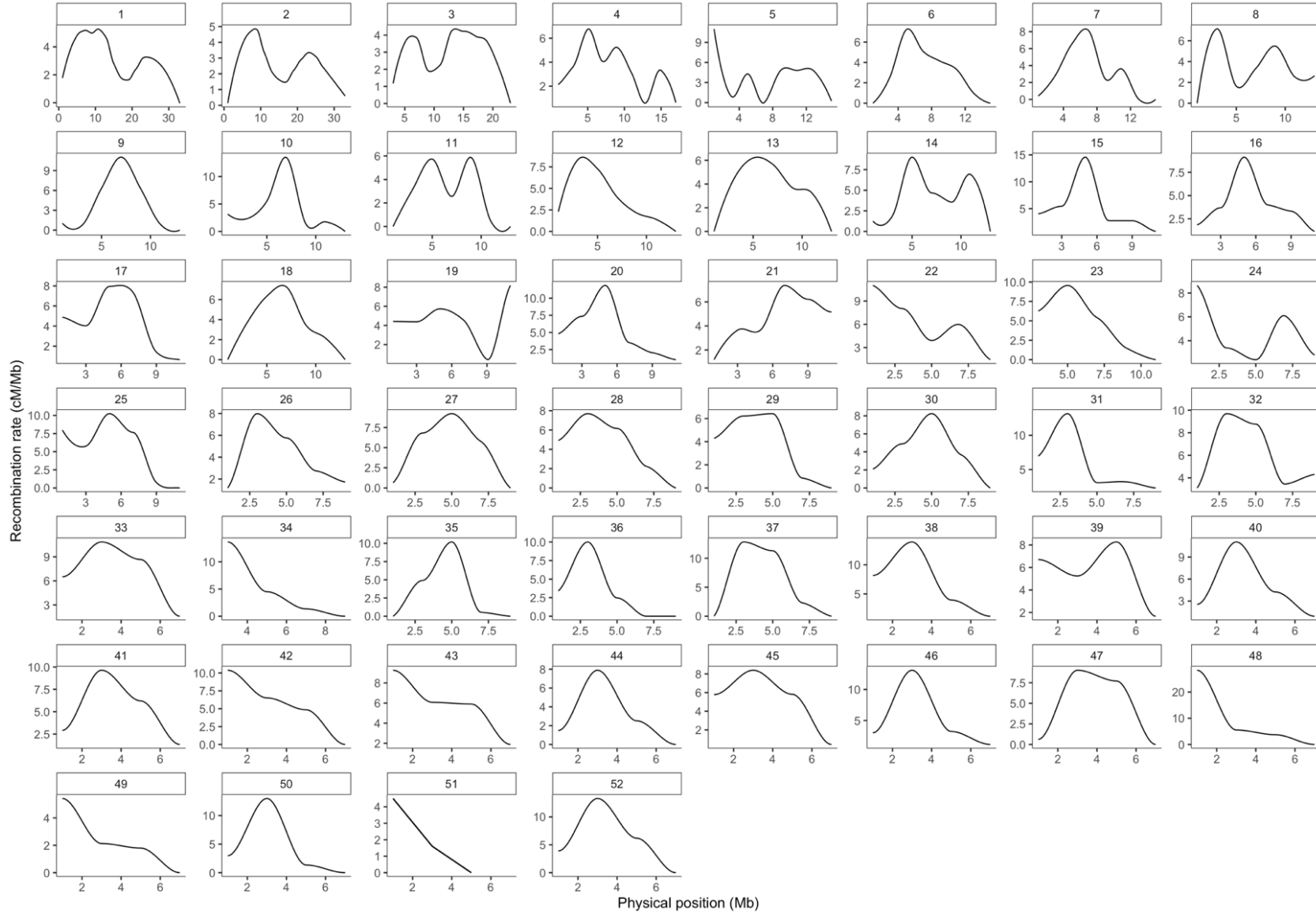

Recombination rate (Swedish)

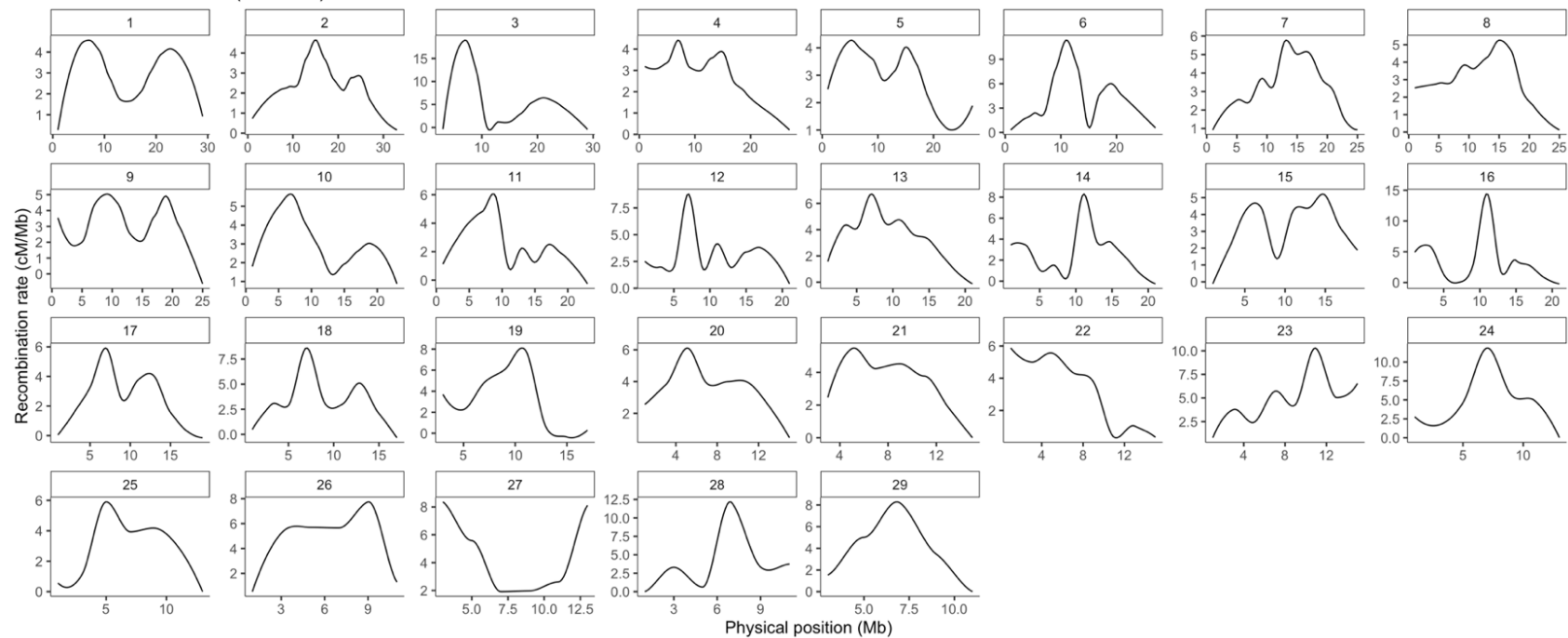

Supplement: S2 Fig — Regional recombination rates (cM, y-axis) as estimated in 2 Mb windows using local regression (LOESS) along the chromosomes in the Catalan and the Swedish population, respectively. Physical position is given on the x-axis (Mb). (PDF) [file pgen.1010717.s002.pdf]

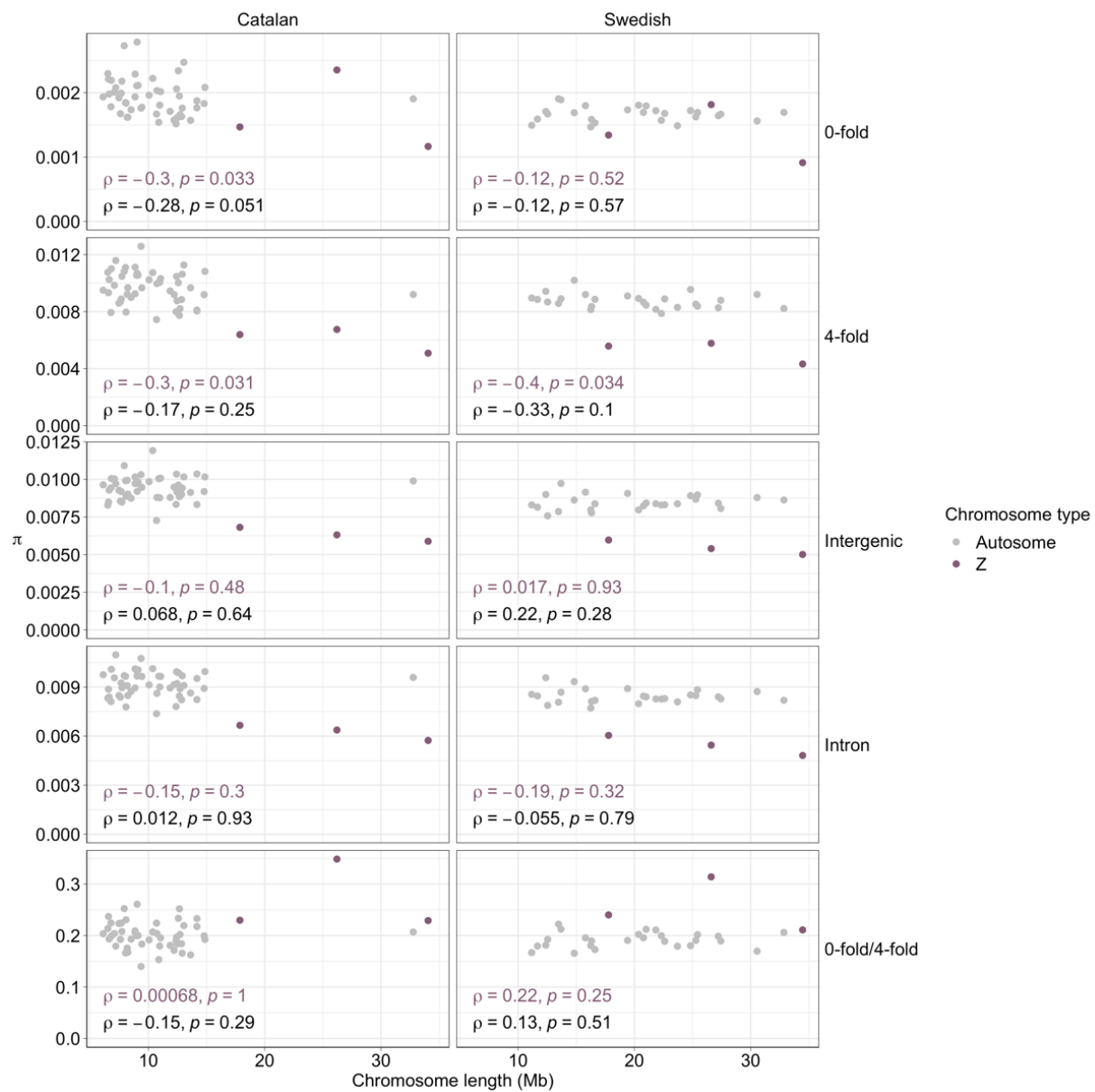

Supplement: S3 Fig — Population specific pairwise differences (π and bottom panel π0 / π4) per chromosome (y-axis) and chromosomal length (x-axis) for each diversity class. Spearman’ rank correlation coefficient including Z-chromosomes (purple) and without Z-chromosomes (black). (PDF) [file pgen.1010717.s003.pdf]

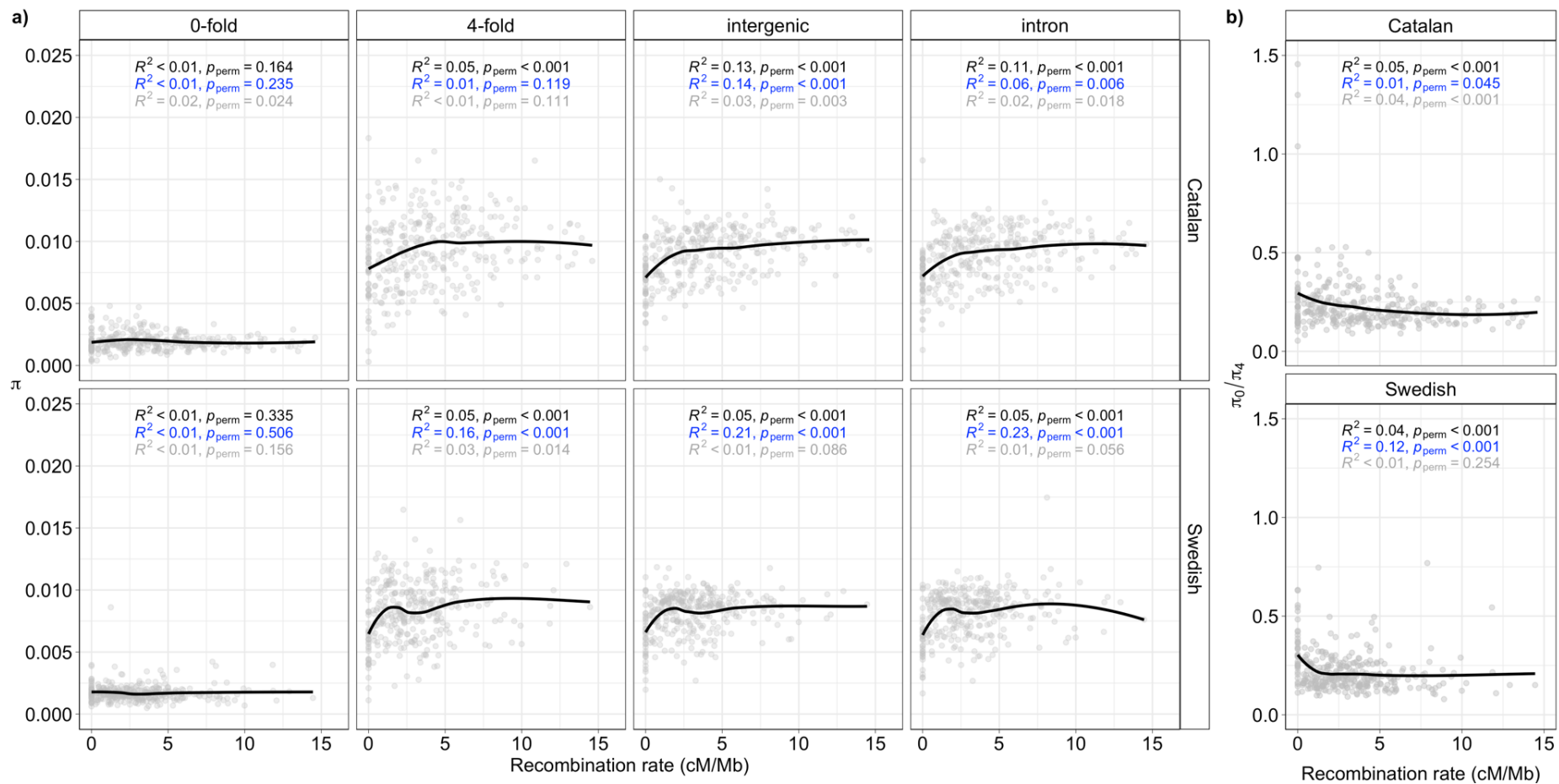

Supplement: S4 Fig — a) Nucleotide diversity (π) as a function of recombination rate in each population estimated in 2Mb windows in zero-fold, four-fold degenerate sites, intergenic and intronic sites for each population. b) The ratio of polymorphisms at 0-fold and 4-fold degenerate sites (π0 / π4) as a function of the recombination rate in each population. Diversity was estimated in 2 Mb windows. Lines represent local regressions, statistics represent results from a model II linear regression, and permuted p-values for the OLS slope are given in black font. The statistics for windows with a recombination rate 2.0 cM / Mb or below and > 2.0 cM / Mb are given in blue and grey font, respectively. (PDF) [file pgen.1010717.s004.pdf]

a)

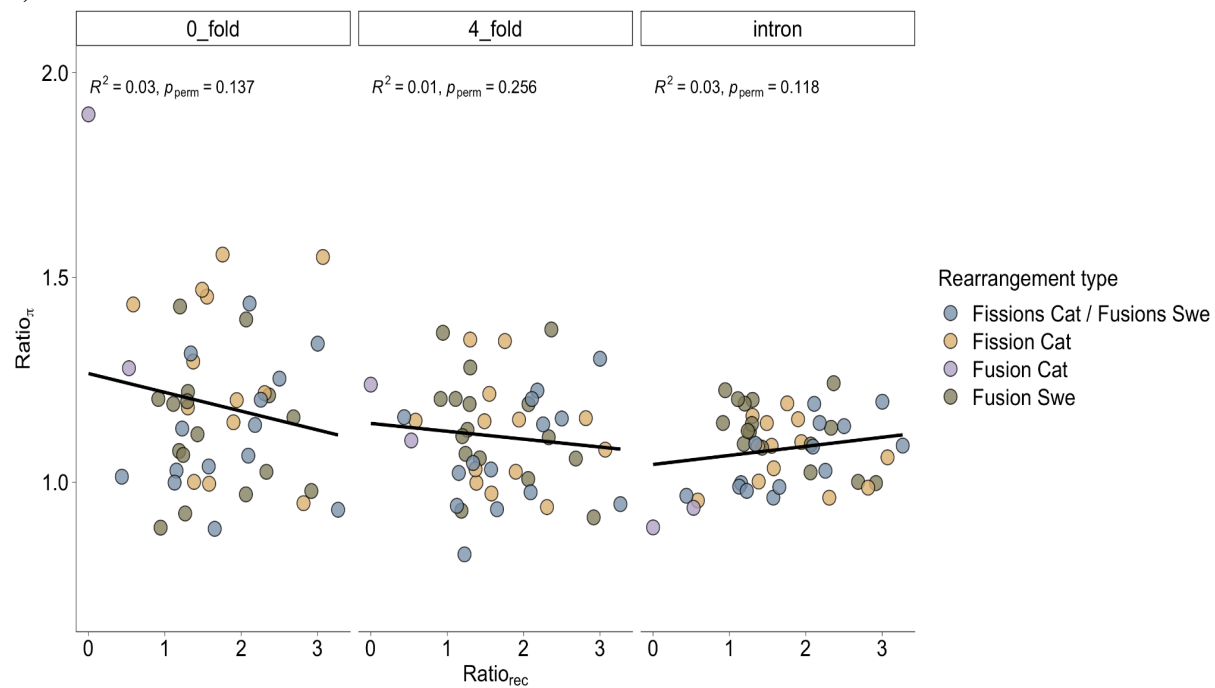

b)

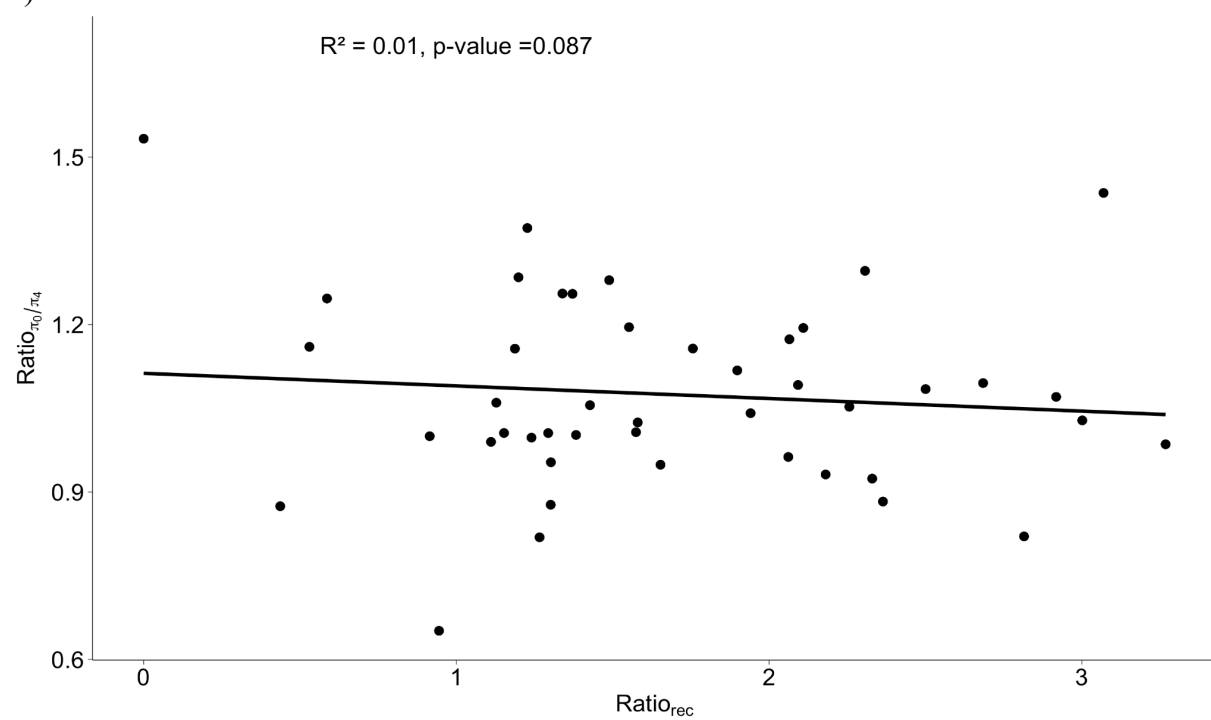

Supplement: S5 Fig — The ratio between Catalan and Swedish estimates of a) diversity (Ratioπ) and b) genetic diversity at 0-fold to 4-fold degenerate sites (Ratioπ0/π4), as a function of the ratio between Catalan and Swedish recombination rate for each ancestral chromosomal unit with known fusion/fission history. The line represents the slope from model II linear regression, with the estimated R2 and permuted p-value. (PDF) [file pgen.1010717.s005.pdf]
