# Supplementary material for: Nascent evolution of recombination rate differences as a consequence of chromosomal rearrangements
Source: PLoS Genet. 2023 Aug 7;19(8):e1010717. doi: 10.1371/journal.pgen.1010717 (PMC10434929; doi:10.1371/journal.pgen.1010717)
Supplement: S1 Table — Mean weighted recombination rate (Rec rate) per chromosome in the Swedish (Swe) and Catalan (Cat) populations. Chromosome length in Mega base-pair (Chr length Mb), genetic map length (cM) and number of markers (Mkr no) per chromosome were also included. (PDF) [file pgen.1010717.s006.pdf]

Pop	Chromosome	Chr type	Chr length (Mb)	Map (cM)	Rec rate	Mkr no
Cat	HiC_scaffold_1	Z	34.04	89.60	2.86	92
Cat	HiC_scaffold_2	Autosome	32.76	79.11	2.42	127
Cat	HiC_scaffold_3	Z	26.20	60.99	2.65	49
Cat	HiC_scaffold_4	Z	17.86	52.82	3.17	51
Cat	HiC_scaffold_5	Autosome	14.86	57.84	4.05	49
Cat	HiC_scaffold_6	Autosome	14.79	46.76	3.21	51
Cat	HiC_scaffold_7	Autosome	14.17	46.94	3.38	49
Cat	HiC_scaffold_8	Autosome	14.17	43.98	3.49	49
Cat	HiC_scaffold_9	Autosome	13.62	53.30	4.45	46
Cat	HiC_scaffold_10	Autosome	13.04	55.40	4.69	44
Cat	HiC_scaffold_11	Autosome	12.90	33.86	2.93	34
Cat	HiC_scaffold_12	Autosome	12.85	52.03	4.10	54
Cat	HiC_scaffold_13	Autosome	12.72	47.60	3.82	50
Cat	HiC_scaffold_14	Autosome	12.66	55.19	4.59	49
Cat	HiC_scaffold_15	Autosome	12.57	54.71	4.74	56
Cat	HiC_scaffold_16	Autosome	12.46	42.32	3.56	54

Cat	HiC_scaffold_17	Autosome	12.42	50.12	4.39	55
Cat	HiC_scaffold_18	Autosome	12.37	44.89	4.25	59
Cat	HiC_scaffold_19	Autosome	12.20	49.99	4.43	50
Cat	HiC_scaffold_20	Autosome	11.86	54.52	4.95	47
Cat	HiC_scaffold_21	Autosome	11.04	46.20	4.25	49
Cat	HiC_scaffold_22	Autosome	10.97	51.54	5.87	43
Cat	HiC_scaffold_23	Autosome	10.89	41.27	4.84	47
Cat	HiC_scaffold_24	Autosome	10.73	36.19	4.11	36
Cat	HiC_scaffold_25	Autosome	10.69	56.05	5.77	46
Cat	HiC_scaffold_26	Autosome	10.37	38.89	4.04	30
Cat	HiC_scaffold_27	Autosome	10.06	44.53	5.05	36
Cat	HiC_scaffold_28	Autosome	9.43	41.68	4.51	36
Cat	HiC_scaffold_29	Autosome	9.37	33.10	4.16	26
Cat	HiC_scaffold_30	Autosome	9.11	34.03	3.93	44
Cat	HiC_scaffold_31	Autosome	9.03	46.33	5.50	21
Cat	HiC_scaffold_32	Autosome	8.99	52.36	6.17	40
Cat	HiC_scaffold_33	Autosome	8.86	52.88	7.02	29
Cat	HiC_scaffold_34	Autosome	8.85	37.28	5.08	23

Cat	HiC_scaffold_35	Autosome	8.51	35.71	4.32	38
Cat	HiC_scaffold_36	Autosome	8.23	28.21	3.70	24
Cat	HiC_scaffold_37	Autosome	8.18	46.37	6.13	17
Cat	HiC_scaffold_38	Autosome	8.07	47.72	7.01	29
Cat	HiC_scaffold_39	Autosome	8.03	39.70	5.56	34
Cat	HiC_scaffold_40	Autosome	7.92	35.13	5.00	30
Cat	HiC_scaffold_41	Autosome	7.72	43.01	6.27	35
Cat	HiC_scaffold_42	Autosome	7.66	34.05	5.23	27
Cat	HiC_scaffold_43	Autosome	7.59	39.82	5.87	20
Cat	HiC_scaffold_44	Autosome	7.46	29.04	4.04	28
Cat	HiC_scaffold_45	Autosome	7.19	34.04	5.20	25
Cat	HiC_scaffold_46	Autosome	7.07	38.18	5.97	23
Cat	HiC_scaffold_47	Autosome	6.81	35.68	5.70	29
Cat	HiC_scaffold_48	Autosome	6.78	40.22	7.43	24
Cat	HiC_scaffold_49	Autosome	6.62	13.37	2.38	13
Cat	HiC_scaffold_50	Autosome	6.56	29.80	4.77	27
Cat	HiC_scaffold_51	Autosome	6.51	10.09	1.95	16
Cat	HiC_scaffold_52	Autosome	6.11	35.73	6.20	34

Swe	HiC_scaffold_1	Z	34.46	83.86	2.92	69
Swe	HiC_scaffold_2	Autosome	32.84	74.13	2.30	104
Swe	HiC_scaffold_3	Autosome	30.54	73.76	2.51	75
Swe	HiC_scaffold_4	Autosome	27.42	64.58	2.51	76
Swe	HiC_scaffold_5	Autosome	27.21	74.66	2.82	76
Swe	HiC_scaffold_6	Z	26.59	77.79	3.05	59
Swe	HiC_scaffold_7	Autosome	25.42	72.77	2.94	79
Swe	HiC_scaffold_8	Autosome	25.27	65.87	2.62	66
Swe	HiC_scaffold_9	Autosome	24.82	66.09	2.69	50
Swe	HiC_scaffold_10	Autosome	23.70	69.18	2.96	88
Swe	HiC_scaffold_11	Autosome	22.60	55.60	2.54	67
Swe	HiC_scaffold_12	Autosome	22.31	67.24	3.10	77
Swe	HiC_scaffold_13	Autosome	21.84	69.45	3.41	75
Swe	HiC_scaffold_14	Autosome	21.00	56.89	2.84	53
Swe	HiC_scaffold_15	Autosome	20.77	57.96	3.02	65
Swe	HiC_scaffold_16	Autosome	20.35	59.78	3.09	45
Swe	HiC_scaffold_17	Autosome	19.41	44.06	2.32	68
Swe	HiC_scaffold_18	Z	17.77	52.45	3.20	36

Swe	HiC_scaffold_19	Autosome	16.60	54.27	3.38	55
Swe	HiC_scaffold_20	Autosome	16.31	51.82	3.30	52
Swe	HiC_scaffold_21	Autosome	16.24	42.30	3.40	37
Swe	HiC_scaffold_22	Autosome	15.79	49.82	3.47	41
Swe	HiC_scaffold_23	Autosome	14.82	57.34	4.10	29
Swe	HiC_scaffold_24	Autosome	13.68	59.45	4.66	49
Swe	HiC_scaffold_25	Autosome	13.47	32.83	2.56	43
Swe	HiC_scaffold_26	Autosome	12.54	50.93	4.62	50
Swe	HiC_scaffold_27	Autosome	12.38	42.68	4.07	22
Swe	HiC_scaffold_28	Autosome	11.66	47.73	4.43	26
Swe	HiC_scaffold_29	Autosome	11.17	35.70	3.44	28
